# Supplementary material for: Are carbon emissions trading and green financial instruments synergistic? -Comprehensive quantitative research based on content analysis
Source: PLoS One. 2024 Mar 7;19(3):e0298601. doi: 10.1371/journal.pone.0298601 (PMC10919601; doi:10.1371/journal.pone.0298601)
Supplement: S1 Data — (ZIP) [file pone.0298601.s001.zip › Data/地级市Z_F工作报告.zip!/╦─┤¿/╤┼░▓╩╨/└┤╫╘╣1⁄2╓┌║┼í╛┬φ┐╦╩2╛▌═°í┐_2018.docx]

各位代表：

　　现在，我代表市人民政府，向大会报告工作，请予审查，并请市政协委员提出意见。

　　2017年工作回顾

　　2017年是本届政府的开局之年。一年来，在省委、省政府和市委的坚强领导下，在市人大、市政协的监督支持下，我们深入学习贯彻习近平新时代中国特色社会主义思想，认真落实习近平总书记对芦山地震灾区作出的“继续发力”“更好发展”等重要指示和省两个“10号文件”精神，牢牢把握稳中求进工作总基调，坚持新发展理念，主动作为、奋力拼搏，较好地完成了市四届人大一次会议确定的目标任务，推进全市经济平稳健康发展和社会和谐稳定。

　　市域经济取得新发展。面对动力接续不足、资源环境约束趋紧等困难和挑战，全力以赴促进经济增长，实现地区生产总值602.77亿元、增长8%，地方一般公共预算收入34.84亿元、增长9.5%，城乡居民人均可支配收入分别增长8.7%、9%。项目投资稳步推进。扎实开展“项目年”工作，全社会固定资产投资完成461.24亿元，其中433个市以上重大项目完成投资285亿元，中恒天汽车、铜头引水等一批重大项目加快建设，川九襄商贸综合体、川西机器异地搬迁等重点工程基本建成。实体经济不断壮大。出台降低实体经济企业成本27条措施，新增亿元企业12户，规模以上工业、服务业企业分别增加26户、32户，规模以上工业产品产销率达到94.1%。县域经济平稳发展。县区年度发展指标基本完成，天全县被评为全省县域经济发展先进县、全省县域经济发展模范县，汉源县被评为全省“三农”工作先进县，名山区被评为全省重大农村改革任务推进示范县(区)。全市民营经济增加值增长9.1%。

　　产业转型取得新成绩。始终将转型发展、优化产业结构作为经济改革与发展主线。有效供给不断增加。深化供给侧结构性改革，“地条钢”等非法产能项目依规取缔，煤矿、非煤矿山等行业落后产能有序关退，清理关停“散乱污”企业144户，关闭小水电企业18户，淘汰16户企业落后产能，单位GDP能耗圆满实现省定目标。创新驱动有效推进。建安工业、雅化集团入选全省军民融合大企业大集团培育名单。实施市级以上重大科技成果转化项目8个，23项科技成果获省政府奖励、位居全省第二，高新技术产业产值增长41.1%。产业结构不断优化。规模以上工业增加值增长9%，完成技改投资55.13亿元，工业集中度达到70.5%，一名微晶、贡嘎雪入选2017年工信部第一批绿色工厂示范名单。服务业增加值增长9.2%，社会消费品零售总额增长11.2%。农业特色优势产业基地面积新增3.6万亩，绿色(有机)认证面积新增14万亩，“蒙顶山茶”荣获“2017中国十大茶叶区域公用品牌”。三次产业结构调至13.4∶47.2∶39.4，经济质量进一步提升。

　　绿色发展迈出新步伐。坚定走“绿而美、绿变金”的发展振兴之路，生态文明建设持续推进。环保整治成效显著。持续打好污染防治“三大战役”，2017年下半年空气质量达标天数比2016年同期增加11.5%，建立市、县、乡、村四级河长制，国控、省控出境断面水质达标率100%，农村面源污染治理有效推进。中央环保督察交办信访件整改完成率87.7%，省环保督察反馈问题整改完成率92.3%，国省挂牌督办问题整改全面完成。生态优势持续巩固。全面开展绿美雅安“七大行动”，完成营造林53.3万亩、道路绿化美化589.3公里，森林覆盖率达到64.77%，城市建成区绿化覆盖率达到42.73%。绿变金逐步显现。深入推进国家全域旅游示范区创建，碧峰峡、喇叭河等景区提升项目加快建设，蒙顶山景区回购顺利推进，一批精品文化主题酒店和民宿景点建成运营，全市旅游综合收入增长22.6%。新增省级森林康养基地8个，4个乡镇入选首批省级森林小镇、位居全省前列。

　　脱贫攻坚取得新胜利。坚持把脱贫攻坚作为头等大事来抓，实现105个贫困村退出、9171名贫困人口脱贫，贫困发生率下降到0.7%。精准扶贫成效明显。统筹实施209个项目，整合资金23.82亿元精准投向贫困地区。完成732户贫困户危房改造，1.2万名建档立卡贫困家庭学生得到资助，救助贫困患者11.2万人次，将1.14万名建档立卡贫困群众纳入最低生活保障范围。脱贫基础不断夯实。在贫困村新发展和改造提升农业特色产业15.7万亩，培育447户科技扶贫示范户。贫困家庭实现就业7332人，开发公益性岗位安置贫困群众2043名。长效机制日渐完善。落实驻村帮扶机制，管好用好扶贫“四项基金”，加强督导考核问责，定期开展“回头看”“回头帮”。扎实开展感恩奋进教育，贫困群众脱贫奔康的内生动力得到进一步激发。

　　发展条件取得新改善。统筹城乡建设，深化改革开放，发展环境不断优化，发展活力不断激发。城乡建设深入推进。完成《雅安市城市总体规划(2013—2030)》实施评估，南环线中段、熊猫绿岛公园、万达广场等一批项目建成投用。成功创建四川省第四届文明城市，芦山、宝兴、荥经、汉源四县成功创建四川省第四届文明县城。宝兴、汉源、石棉三县成功创建国家卫生县城。雨城区多营镇、宝兴县灵关镇分别成功创建国家级、省级特色小镇。全市常住人口城镇化率提高1.4个百分点。新建幸福美丽新村300个，创建省级“四好村”63个。基础设施不断完善。雅康高速公路雅泸段建成通车，川藏铁路成雅段、峨汉高速公路、雅安火车站加快建设。成雅快速通道、多营青衣江大桥开工建设，改建和提升农村公路409公里。锅浪跷、出居沟等水电站加快建设，4个110千伏输变电工程竣工投运。改革开放成效明显。统筹推进20项国家和省级部署改革试点、13项市级改革试点，农村、国资国企、医药卫生等改革取得新进展。组织参加中外知名企业四川行、西博会进出口商品展等重大投资促进活动，全年引进到位市外资金375.4亿元、增长5.1%。

　　民生事业取得新进展。深入践行以人民为中心的发展思想，一批惠民政策和举措落地见效，人民生活不断改善。民生实事大力实施。一般公共预算民生支出占比达到66%，十项民生工程和20件民生实事全部完成。重建成果巩固提升。除16个国外贷款项目外重建项目全面收官。着力强化重建成果的运营管理，总规项目使用率达到99%以上，重建效应明显显现。社会保障更加完善。大力实施全民参保计划，全面整合城乡居民医疗保险制度，实现跨省异地就医住院费用直接结算。城乡特困人员供养标准提标。完成危旧房棚户区改造4915套。城镇登记失业率3.88%。公共服务提质扩面。“三免一补”工程扶助学生15.3万人次，高考本科上线率达到47%。雅安天立学校一期建成开学，现代学徒制试点加快推进。市妇女儿童中心医院、市儿童福利院建成投用，乡镇卫生院、社区卫生服务中心住院诊疗量分别增长15%、23.4%。新建全民健身路径41条，承办省级以上体育赛事活动14项(次)。切实做好妇女、儿童、老龄、残疾人、社会福利、慈善、红十字、双拥等工作，防震减灾、统计、气象、档案、保密、科协、地方志、社会科学、外事、侨务和港澳台等工作扎实推进。

社会治理取得新进步。创新社会治理，切实防范各类风险，社会大局保持和谐稳定。法治雅安加快建设。大力开展“七五”普法，法治扶贫“五个一”工作机制在全省推广学习。圆满完成第十届村(居)民委员会换届选举。打击违法犯罪成效明显，刑事立案数下降25.2%、破案率提升6.5个百分点，实现年度命案破案率100%。矛盾纠纷有效化解。建立矛盾纠纷多元化解机制，妥善化解了一批涉军、库区移民、征地搬迁等领域不稳定因素，各类矛盾纠纷调解成功率达到95.3%。社会风险得到防控。大力开展互联网金融风险专项排查，依法打击非法集资。安全事故死亡人数、直接经济损失分别下降25.5%、31.6%，安全形势稳定向好。开展学校食堂、地沟油、医疗器械使用等专项整治，食品药品监管得到加强。强化应急联动和监测预警，应急处突能力进一步提升。国家安全、民族、宗教工作有序推进，国防动员和民兵预备役工作得到加强。

政府建设取得新成效。持之以恒转变政府职能和工作作风，政府执行力、公信力和依法行政能力不断提升。依法行政扎实推进。自觉接受市人大及其常委会的法律监督、工作监督和市政协的民主监督，认真办理人大代表建议135件、政协委员提案249件，办结率100%。完善重大行政决策程序，推进行政权力和“两法衔接”信息共享平台建设。行政诉讼案件下降20.5%。简政放权持续深入。取消和调整行政许可事项9项，深入推进全省相对集中行政许可权改革试点，实行重点投资项目代办制度，建成一体化政务服务平台。全面实施“多证合一”，新登记企业2718户。廉政建设不断加强。严格执行中央八项规定精神、国务院“约法三章”和省委省政府十项规定，全市“三公”经费下降20.2%。政风行风群众安全感、满意度测评名列全省前茅，政府系统党风廉政建设和反腐败斗争取得新进展。

　　过去一年的成绩来之不易，本届政府工作开局良好。这是党中央、国务院，省委、省政府和市委坚强领导的结果，是市委统揽全局、科学决策的结果，是市人大、市政协强化监督、协同支持的结果，是全市干部群众同心同德、奋力拼搏的结果。在此，我代表市人民政府，向全市人民，向人大代表、政协委员，向各民主党派、工商联、无党派人士、各人民团体和社会各界人士，向驻雅解放军、武警官兵、政法干警和民兵预备役人员，向所有关心支持雅安发展的朋友们，致以真诚的谢意!

　　在报告工作成绩的同时，我们也清醒地认识到，当前全市发展还面临着一些问题和困难：重大项目支撑乏力，特别是去年受客观因素影响，一批项目停建缓建，市四届人大一次会议确定的全社会固定资产投资目标未达预期;面对新时代新要求，产业转型升级压力较大，实体经济发展后劲不足;经济质量有待提高，产业结构需进一步优化;城镇发展内生动力不足;财源建设有待进一步强化;民生事业离人民群众愿望还有差距;少数干部思想解放不够、推进发展敢于作为意识不强，机关效能还需进一步提升。对此，我们将直面问题、勇于担当、攻坚克难、着力解决，绝不辜负全市人民的期望。

　　2018年工作安排

　　党的十九大作出了中国特色社会主义进入新时代的重大政治论断，确定了决胜全面建成小康社会、开启全面建设社会主义现代化国家新征程的宏伟布局。省委十一届二次全会进一步丰富完善了治蜀兴川总体工作格局，作出了“一个愿景、两个跨越、三大发展战略、四项重点工程”战略谋划。市委四届三次全会确立了“1485”总体发展思路，为雅安经济社会发展描绘了美好蓝图。进入新时代，迈向新征程，这是我们做好新时代政府工作的基本遵循和行动指南。

　　2018年是贯彻落实党的十九大精神的开局之年，是雅安实现“五年整体跨越”目标的决胜之年。做好今年工作，意义十分重大。今年政府工作的总体要求是：以习近平新时代中国特色社会主义思想为指导，深入贯彻落实党的十九大精神，认真贯彻落实党中央、国务院，省委、省政府的战略决策和市委工作部署，始终保持专注发展转型发展定力，坚持稳中求进工作总基调，坚持新发展理念，紧扣社会主要矛盾变化，按照高质量发展要求，认真落实市委“1485”总体发展思路，统筹推进稳增长、促改革、调结构、惠民生、防风险各项工作，打好防范化解重大风险、精准脱贫、污染防治三大攻坚战，抓重点、攻难点，开拓奋进、真抓实干，努力推进绿色发展振兴，确保实现“五年整体跨越”目标。

　　今年全市经济社会发展主要预期目标建议：地区生产总值增长8%;规模以上工业增加值增长9.5%;社会消费品零售总额增长10%;地方一般公共预算收入增长8.5%;城乡居民人均可支配收入分别增长8.1%、9.1%;城镇登记失业率控制在4%以内;环境保护和节能减排完成省定目标任务。

　　为确保实现以上目标，我们将重点抓好九个方面工作：

　　一、突出投资拉动，推动经济较快增长

　　投资拉动仍是当前推进全市经济发展的主动力，项目始终是经济工作的主抓手。认真落实市委“项目攻坚年”工作要求，强基础、优机制、快建设，千方百计确保全社会固定资产投资增长12%，推动经济较快增长。

　　提升项目策划包装和落地能力。进一步强化项目前期工作，站高谋远，谋划一批大项目、好项目。成立市项目编研中心，组建项目包装储备专班队伍，切实加强高端项目策划和大项目包装、储备工作。精准对接国家和省重大发展战略与产业发展方向，努力争取一批项目进入国省规划盘子。提升市、县区项目策划包装储备联动水平，建立项目工作策划包装与落地建设一体化电子跟踪信息平台，提高项目工作能力与项目落地实效。力争常态化储备项目4000个、估算投资8000亿元以上，其中5亿元以上项目360个以上、10亿元以上项目150个以上。

　　强力推进招商引资。把招商引资作为项目工作重大抓手，主动出击、高位推进。在北京、成都、杭州、深圳设立市县联动、专班专责、绩效激励的招商分局，鼓励各部门各行业奋勇争先招商引资，千方百计提升招商引资实效。进一步完善优惠政策，突出产业和新技术等招商重点，带动以商招商、园区招商、委托招商和互联网招商，力促一批重点招商项目落地雅安。严守绿色发展底线，从严防范招商项目泥沙俱下。力争年内引进到位市外资金395亿元以上，投资5亿元以上、10亿元以上产业项目分别达到9个、3个。

　　突出大项目建设。将大项目引进和建设作为投资拉动的主攻方向。围绕“八大行动”谋划一批大项目，坚持双眼向外向上争取新上一批大项目。健全领导干部挂联大项目机制，建立完善台账管理、巡查督查等制度，切实加快大项目落地和建设进度。坚持抓大促小，突出重点项目和产业项目，全市实施亿元以上项目190个、5亿元以上项目70个、10亿元以上项目35个，完成年度投资290亿元以上。其中，产业项目亿元以上90个、5亿元以上40个、10亿元以上20个，完成年度投资130亿元以上。

　　二、强化旅游促动，推进生态文旅融合发展

　　雅安生态文化旅游资源丰富，生态文旅融合发展潜力巨大。面对生态功能区建设和从严环保新形势，坚决落实好市委“全域旅游”“绿美生态”行动工作要求，走“绿而美、绿变金”的发展振兴之路。

　　强力打造雅安特色文旅品牌。落实党中央、国务院关于传承发展中华优秀传统文化的要求，深入挖掘雅安文化资源，推动生态资源、文化资源转化为旅游优势。以雅安生态为本底，传承大熊猫、茶马古道、蒙顶山茶、雅安藏茶、荥经黑砂等特色文化根脉，全力打造大熊猫、茶马古道两大国际文化品牌。突出一县一品的地方特色文化旅游景区，协同开发以安顺场、夹金山为重点的红色旅游线和三雅、西康、汉文化等地方特色文化景点。强化文化资源与旅游资源的深度融合，打造大熊猫文化国际特色旅游目的地和世界茶马古道精品旅游走廊，以此串联全市旅游景区，逐步形成“两藤环绕、藤上结瓜、多点开花”全域旅游发展的雅安模式。

　　力补旅游发展三个短板。面向市场，审时度势，加快由重景区数量向提升景区质量转变、单纯自然景观开发向生态文旅深度融合转变、县区各自散打向抱团联动转变。力补缺乏龙头景区的短板。着力推进碧峰峡、蒙顶山、喇叭河等已有景区升级，加快南丝路·黑砂都、王岗坪等新兴景区建设，积极争取好莱坞(中国)茶马古道国际旅游度假区、未来嘉年华雅安国际旅游度假区、周公山旅游度假区、十里梅香·茶源小镇等重大文旅项目能落地开建，大力促进龙头景区和旅游目的地建设。力补要素配置不完善的短板。全面提升吃、住、行、游、购、娱等旅游要素建设与管理水平，加快建设一批高星级精品酒店，推进旅游主通道和支线道路建设，新建和改扩建旅游厕所112座，完善标识标牌、停车场等旅游公共服务设施。力补旅游营销不良的短板。推进市县区旅游销售一盘棋，市、县区与旅游企业共同筹资推进向外联动促销。引进和联络一批旅行社，开通旅游客运专线，大手笔策划、多维度推介，切实提升旅游营销实效。围绕促进农民增收、增加财政贡献、拉动相关产业发展等重点指标，完善旅游工作考核体系，切实推进旅游经济健康发展。

强化康养产业发展。优化市域康养产业发展规划布局，切实防范县区康养产业同质化布局与发展。有效利用好国家医养结合试点政策，切实推进一批医养结合示范项目建设。突出园区抓手，推进龙苍沟森林康养度假区、百丈湖旅游度假区、石棉温泉康养城等项目建设尽早见效，启动碧峰峡熊猫山谷生态康养区、茶马古道清溪古城旅游度假区、夹金山青衣江源森林康养度假区等康养项目规划和建设，年内初步建成较大规模或具示范意义的康养园区(基地)3个以上。大力发展民宿旅游，市域错位发展森林康养、阳光康养、医疗康养、温泉康养、运动康养、旅居养老，协调推进康养、医养配套建设，加快将雅安打造成西蜀生态康养目的地步伐。

　　持续增强绿美本底。坚持从严从实，严守生态保护红线，坚定不移地保护好祖先留给雅安人民的宝贵绿色财富。深入实施绿美雅安“七大行动”，完成营造林41万亩，治理水土流失面积15万亩，确保全市森林覆盖率达到65%。启动创建省级森林城市，改造提升城市绿地，建设城郊森林公园、湿地公园5个，中心城区人均公园绿地面积达到11平方米。加强生物多样性保护，积极推进大熊猫国家公园雅安片区规划建设。坚持科学合理保护与利用并重，千方百计强化绿变金实效。

　　三、加快产业转型升级，推动经济扩容提质

　　积极适应国家由高速发展阶段向高质量发展阶段转变的新时代经济发展要求，深化供给侧结构性改革，大力推进创新驱动，积极培育实体经济产业，不断推进全市经济扩容提质。

　　强化飞地经济发展。将实施“飞地经济”行动作为产业接续的关键抓手。进一步创新体制机制，加大向县区倾斜政策力度，完善考核激励办法，调动县区联动和参与发展飞地经济的积极性。鼓励县区与飞地园区共同出资成立开发运营公司，推动县区招引入园单个项目股份合作化经营管理，以市场法则共谋飞地园区协同发展。加快园中园合作共建力度，激励相关县区引进工业项目落户飞地园区。强化基础设施建设，拓展提升物流园区规划面积并加快建设，确保物流园区主体工程与铁路货运站同步投用。完善公共服务平台，推行行政审批“跟章制”或委托“一章制”，探索飞地园区管理服务“一体化”模式。强化飞地园区招商，力争引进项目10个、到位资金26亿元以上。全力做好飞地园区升级国家级经济技术开发区培育与迎检工作。协同发展县区工业园区，积极吸引甘孜等地合作发展飞地经济。力争飞地园区营业收入达到360亿元，全市工业园区营业收入突破850亿元。

　　大力发展特色优势产业。实施“千亿产业”行动，分产业制定发展规划和实施方案，务实推动经济快速高质量发展。加快中恒天汽车实现量产，推动川西机器异地搬迁项目建成投产，力争汽车及机械装备制造产业总产值由65亿元增加到75亿元。加快夯鑫玄武岩、东辰科技等项目落地建设，推进一名微晶、中雅三期等技改扩能，提升四环锌锗、俊磊科技有色金属产品市场竞争力，做大四川亿欣、贡嘎雪等企业规模，力争新材料产业总产值由107亿元增加到130亿元。科学推进水电资源综合开发利用，加快汉源、石棉等地风电、太阳能发电项目布局，力争清洁能源产业总产值由144亿元增加到155亿元。做好茶叶企业整合与做大做强工作，提升已有农产品加工龙头企业规模与质量，下功夫发展一批雅药、雅竹、雅果等新兴农林加工企业，力争农产品加工产业总产值由95亿元增加到105亿元。统筹推进物流、商贸、电子商务等融合发展，力争服务业增加值由237亿元增加到257亿元。坚持稳商与招商并重，大力支持现有企业发展壮大，力争年内规模以上工业、服务业企业分别新增20户、25户。推动国有企业做大做强，力争市属国有企业营业收入、利润分别增长10%、14%。

　　推动创新驱动发展。主动参与成德绵全面创新改革试验，加快融入攀西战略资源创新开发试验区项目建设。推进军民融合发展，力争建安、川西等企业军民融合项目建成投产。加强科技创新和成果转化，建设省级工程技术研究中心1个，高新技术产业产值超过150亿元，实施市级以上重大科技成果转化项目10个，科技进步贡献率提高1个百分点。大力推进大众创业、万众创新，新增创新创业载体2个，孵化支持各类创新创业主体或项目30个。与省级部门、高校联合建设雅安藏茶研究院、荥经砂器研究院，强化四川省碲铋产业研究院建设。创新人才激励机制，实施“雅州英才”工程，不断强化人才支撑。培育壮大本地“土专家”队伍，吸引和培养一批涉及砂器、根雕、石雕等具有地方文化特色的大师工作室，加大非遗文化市场融合和实用技能技术推广力度。

　　大力发展县域经济。聘请高端宏观经济研究团队，深化市域经济宏观研究，编制全市县域经济和市域产业发展规划。支持县区向上争取政策、项目和资金，统筹对外开放和招商引资工作，协同推进商贸、文旅和涉农产业跨行政区域资源配置与联动发展。强化考评机制引导，努力提升县域经济在省内的综合评价排位。落实促进民营经济发展政策措施，依法保护民营企业合法权益，营造企业家健康成长环境，推动民营经济大发展，力争民营经济增加值占地区生产总值比重达到60%。

　　切实做好财税金融工作。深化财税体制改革，推进财政事权和支出责任划分。加强财源建设，培植重点税源企业，不能给群众和政府带来效益的事不干，既给群众带来好处又能让政府有收益的事快干多干，努力培育壮大税源和新的经济增长点。积极转变财政支持经济发展方式，加大发展资金筹措力度，大力支持重大项目、重点企业发展。加快普惠金融体系建设，鼓励金融机构强化创新产品服务。推动具备条件的企业上市挂牌融资，完成2家县农村信用联社改制为农村商业银行。新增银行信贷60亿元以上，全域银行贷款余额达到900亿元以上。

　　四、突出城镇带动，全方位推进“东进融入”

　　“东进融入”是雅安未来发展振兴的战略性选择。大力实施“东进融入”行动，以区域规划、基础设施、产业发展等为抓手，加速中心城区东拓，加快融入成都大都市区和成都平原经济区。

　　提升城镇规划管控水平。树立大规划理念，启动《雅安市城市总体规划(2013—2030)》修编，推进旅游、产业、交通、生态等专规融入。切实推进城市详规和各项专业规划编制，加快推进城市设计、城市双修专项规划编制。强化规划刚性管控，加大对规划执行情况的监督检查力度。扩大市规委会评审和管理规划范围，提升规委会权威。建立完善专家评审、重大规划与设计社会公示等制度，对县区重大规划上审一级。实行重大项目规划和重要建筑创意公开征集制度，进一步提升城市规划设计品位。

　　强化中心城区建设。启动并力争完成“雨名飞”交通、产业、组团功能一盘棋规划编制，加快雨名快速通道等基础设施建设，推进“雨名飞”同城化发展。强化大兴新区、草坝片区建设，推进中心城区向东拓展。加快大兴滨江文化绿廊、雅安火车站、雅安国际会展中心等十大城市基础设施项目建设，进一步拉大城市骨架。加快中国供销川西农副产品物流园暨西蜀商展中心、茶马古道藏茶城、雅安希尔顿五星级宾馆等十大城市产业项目建设，着力提升城市产业支撑动能。大力实施危旧房棚户区改造、房地产市场分类调控、背街小巷治理等十大城市民生项目，进一步提升市民城市建设获得感。启动国道351线雨城区姚桥至草坝段、成雅高速公路金鸡关互通综合改造等工程，加快南环线西段和东段建设，进一步提升主城区对外交通融入度。做好道路、路灯、雨污水管网等设施改造、维护和管理，切实增强城市功能配套。稳步推进城市执法体制改革，完善城市管理网格化工作机制，提升城市管理精细化、智慧化、法治化水平。力争中心城区建成区面积新增1.6平方公里。

加快城镇体系建设。强化县城规划、建设和管理，加快市政基础设施和公共服务设施建设，进一步增强县城承载能力和对县域的辐射带动作用，力争6个县城建成区面积新增1.1平方公里以上。加快小城镇建设，突出个性特点和历史文化传承，着力增强服务功能，提升吸纳就业和人口聚集能力，创建省级以上特色小城镇2个。坚持政府投入和市场融资相结合，鼓励社会资本参与新型城镇化建设。完成10个撤乡建(并)镇、9个村改居工作，力争1.5万农业转移人口落户城镇，全市常住人口城镇化率提高1.2个百分点。

　　积极融入成都大都市区。建立政府部门主动对接成都机制，推动基础设施、产业发展、人才交流等务实有效融入。协调推进川藏铁路成雅段竣工投运，加快成雅快速通道建设，积极推进天雅高速公路、雅眉乐自铁路等项目前期规划对接工作，推动成雅交通大联通。对接成都产业发展指导目录和产业转移指导意见，布局配套一批主导产业、关联产业。加快成雅、雅双工业园建设，主动承接一批成都产业转移和投资项目。推动雅安无水港等一批物流项目落地建设，争取引进一批成都物流企业转移雅安，积极打造服务康藏、对接成都的物流“桥头堡”。强化人才交流和培训力度，主动承接成都智力辐射。

　　五、落实乡村振兴战略，推进农业农村现代化

　　国家实施乡村振兴战略是我市发展的重大机遇。认真贯彻落实《中共中央 国务院关于实施乡村振兴战略的意见》，按照“产业兴旺、生态宜居、乡风文明、治理有效、生活富裕”的总要求，大力开展“五美乡村”行动，加快推进农业农村现代化。

　　深化农业农村改革。落实第二轮土地承包到期后再延长30年的政策，依法保护集体土地所有权、稳定承包权、放活经营权，推动土地规范有序流转。加快推进农村产权制度改革，全面推进农村产权“多权同确”，完善农村土地“三权分置”机制。大力发展村级集体经济，支持和发展农业适度规模经营、农村服务业、合作经营等多种形式的村级集体经济。深化供销合作社综合改革，加快基层社建设和服务网络改造，提升农业社会化服务水平。强化农业农村投入保障，建立健全涉农资金统筹整合长效机制。

　　大力发展现代农业。深化农业供给侧结构性改革，坚持建基地、创品牌、搞加工，推动乡村产业美。持续发展壮大特色优势产业，新发展特色产业基地4万亩。全力推进三条百公里百万亩产业带核心区建设，在每条产业带核心区选择1个乡镇、2至3个村开展乡村振兴试点示范。巩固提升国家绿色(有机)农业示范区建设成果，推进农产品质量安全溯源体系建设，新增绿色(有机)认证面积5万亩、“三品一标”农产品10个。新建高标准农田2.7万亩，稳定粮食生产，确保粮食安全。加快“1+8”现代农业产业园区建设，建设一批农业公园和田园综合体。大力培育新型农业经营主体，培育省级农民专业合作示范社7个，新增市级以上龙头企业15个，产业化龙头企业销售收入增长8%以上。大力推进农村一二三产业融合发展，积极培育休闲农业、创意农业、乡村旅游、农村电商等新产业新业态。

　　建设“五美乡村”。在强化乡村产业美的基础上，加强村落民居建设，改善农村人居环境，提升公共服务水平，完善乡村治理体系，推动乡村民居美、环境美、生活美、乡风美。推广“小规模、组团式、微田园、生态化”建设模式，统筹抓好3个全省幸福美丽新村示范县建设，建成幸福美丽新村300个，创建市级“四好村”300个、省级“四好村”60个。全面整治农村生活垃圾、生活污水、水体污染，进一步完善农村生活垃圾“户分类、村收集、镇(乡)转运、县处理”模式，村庄垃圾无害化处理率达到70%;完成80个新村聚居点污水处理设施项目建设，50%的行政村生活污水得到有效处理。统筹抓好交通、饮水、卫生、文化等功能配置，切实满足农村居民生产生活需要。

　　六、加快基础设施建设，持续改善发展条件

　　完善基础设施建设是雅安发展振兴的重要内容。加快建设川西交通枢纽，统筹推进能源、水利、信息等基础设施建设，更好支撑经济社会发展。

　　实施“交通通达”行动。力争开工建设川藏铁路雅康段、泸石高速公路，协同加快峨汉高速公路建设。开工建设省道217线石棉至甘洛段、省道105线雅安至上里段、雅望路雅安城区至周公山温泉段等项目，做好国道318线成雅界至雨城多营段改造提升、国道351线夹金山隧道、省道431线名山经雨城至芦山段等项目前期工作。推进“四好农村路”建设，改善提升农村公路240公里，提高跨行政区域村组路网通达率。提升道路管护水平，实施国省干线大中修61公里，路面使用性能指数达到85。

　　强化能源、水利基础设施建设。加快锅浪跷、出居沟等水电站建设，完成小河220千伏输变电工程前期工作，建成3个110千伏输变电工程。大力实施农网改造升级，切实做好城市配电网建设。推进电力体制和天然气供气体制改革，积极引导企业煤改电、煤改气。加快九龙水库、永定桥大田支渠工程建设，建成铜头引水、南郊水厂引水工程。

　　推进新型智慧城市建设。加快云计算和通信网络等信息基础设施建设，推动5G商用试点工作。建设“宽带中国”示范城市，实施“宽带乡村”工程，力争全市固定宽带家庭普及率由64%提升到70%、行政村通宽带比例由95%提升到99%。加快视联网大数据中心、城市公共信息服务平台、电子政务云等项目实施和推广，加快实现信息共享。

　　七、坚决打好“三大攻坚战”，夯实全面建成小康社会基础

　　精准脱贫、污染防治、防范化解重大风险是中央确定的事关群众利益的工作重点，也是推进科学发展工作中必破的难题，必须毫不放松地抓牢抓实。

　　扎实推进精准脱贫。进一步加大力度、集中攻坚，确保现行标准下剩余的111个贫困村全部退出、8000名贫困人口全部脱贫。完成261户贫困户危房改造和179户贫困户土坯房改造，完善交通、电力、通信等基础设施，落实教育、医疗等扶贫政策，改善群众生产生活条件。继续抓好产业就业扶贫，增强脱贫造血能力。加强思想道德教育和感恩教育，办好农民夜校，引导贫困群众养成好习惯、形成好风气。全面开展“回头看”“回头帮”，着力巩固脱贫成效。完善脱贫攻坚督导考核机制，抓好扶贫领域作风问题专项治理，坚决防止虚假脱贫、数字脱贫。

　　强力推进污染防治。推进空气质量达标行动，深化PM2.5和PM10治理，推广应用新能源汽车，加大餐饮油烟、机动车尾气、建筑扬尘等整治力度。全面落实河(湖)长制，协同做好《雅安市村级河长制条例》地方立法工作;实施最严格的水资源管理制度，深入实施流域综合整治，大力开展饮用水源环境安全保障及良好水体保护行动。推进土壤污染状况详查，开展分类管控和修复。加大城镇污水和城乡垃圾处理设施建设力度，年内完成40个生活污水处理设施项目和9个生活垃圾处理设施项目建设。统筹推进中央、省环保督察发现问题整改，健全完善长效机制。全面开展第二次全国污染源普查工作。继续深化环境网格化监管，对各类环境违法行为坚决“零容忍”从严打击。

防范化解重大风险。加强对地方各类交易场所的监管，积极化解民间融资风险、金融机构不良风险。坚决打击非法集资、金融诈骗等违法违规活动，依法保护人民群众合法权益。防范化解房地产市场风险，建立完善多主体供给、多渠道保障、租购并举的住房制度，严厉查处捂盘惜售、炒卖房号等行为，推进市域房地产市场健康有序发展。稳妥有序化解政府存量债务，坚决避免违规融资担保和超预警线举债行为。

　　八、大力改善社会民生，切实增进百姓福祉

　　不断满足人民群众日益增长的美好生活需要是我们最大的奋斗目标。实施“民生福祉”行动，集中力量办好一批民生实事，大力发展社会事业，努力让改革和发展成果更好惠及全市人民。

　　扎实办好民生实事。抓好十项民生工程，办好20件民生实事，确保一般公共预算民生支出占比66%以上。健全创业带动就业机制，实现城镇新增就业1.6万人。加强多层次社会保障体系建设，深入实施全民参保计划，实现社会保险法定人群全覆盖。完善社会救助体系和困难群众帮扶机制，加大城乡低保兜底力度，深化“量体裁衣”式残疾人服务，协同推进社会福利、慈善等工作。深入实施“菜篮子”工程，中心城区落实常年蔬菜生产基地1.5万亩，建设智慧菜市场5个以上。进一步抓好民族乡镇、中高山地区发展和水电库区后续建设，推动与全市同步致富奔康。

　　推动文化事业产业繁荣发展。弘扬社会主义核心价值观，强化市民思想道德建设。对接省文化事业产业发展规划，强化文化服务体系，推进文化旅游融合，壮大新兴文创产业，繁荣社会主义文艺和社会科学，积极推进文化产业今后逐步成为全市支柱性产业。加强文物保护利用和文化遗产保护传承，建设中国茶马古道博物馆、中国藏茶展览馆、西蜀民俗展示馆，鼓励社会力量创办民间博物馆。深化文化体制改革，支持社会资本参与公共文化建设。探索建立文学艺术创作基地，引进和培养文化专业人才，激励创作一批能在全国全省有影响力的文艺作品和文化精品。建立健全公共文体场馆错时开放制度，做好基层综合文化活动室信息工程建设。联合国内省内相关机构进一步办好蒙顶山茶文化旅游节，积极申办中国茶马古道国际文化旅游节，办好雅安首届熊猫灯会等大型群众文化活动，丰富人民群众文化生活，提升雅安城市影响力和知名度。积极策划和启动中国世界诗歌文化康养园建设，推进文艺、音乐、网游、体育等多元素与游、养、娱融合发展。

　　统筹发展社会事业。优先发展教育事业，扩大学前教育资源，新建幼儿园5所，新增普惠性学位2260个;推进义务教育均衡发展，开工建设雨城区金安学校、石棉向阳教育园区学校，着力化解“择校热”“大班额”等问题;实施高中教育普及攻坚计划，将名山一中创建成省一级示范高中;加快发展现代职业教育，全力支持优质民办教育，着力办好特殊教育和继续教育;优化教育资源配置，强化师德师风建设。加快健康雅安建设，构建智慧医疗服务体系，建设全市远程医疗中心，开展家庭医生签约服务，推进中医药传承和创新。全面发展妇女、儿童、老龄、体育、红十字等事业，切实做好防震减灾、气象、档案、保密、科协、地方志、社会科学、外事、侨务和港澳台等工作。

　　九、加强和创新社会治理，巩固安定团结社会局面

　　社会和谐稳定是经济良好发展的重要条件，也是人民群众安居乐业的共同心愿。加快推进社会治理体系和能力现代化建设，努力打造共建共治共享的现代化社会治理格局。

　　完善社会治理体系。深入推进依法治市，全面推进“七五”普法，健全覆盖城乡的公共法律服务体系，巩固提升法治扶贫“五个一”工作机制。以公安110指挥中心为基础平台，以规范化综治中心建设为抓手，整合公安天网工程、农村“雪亮工程”和部门公共大数据与信息源平台，构建一体化的“1+N”资源共享、融合联动的社会治理模式。加强社区服务中心(站)建设，支持群团组织参与社会治理。落实《雅安市新村聚居点管理条例》，完善村务公开、民情恳谈、民主管理等制度。加强社会信用体系建设，建立守信联合激励和失信联合惩戒制度。加强民族宗教工作。强化国防和后备力量建设，促进军地互动发展、军民融合共建。

　　深化平安雅安建设。全面落实习近平总书记总体国家安全观，深入开展反渗透、反分裂、反恐怖、反邪教斗争。强化社会治安防控和公共安全体系建设，依法严厉打击黄赌毒和各类违法犯罪活动。深入开展扫黑除恶专项斗争，坚决扫除雅州大地上的一切涉黑涉恶势力。深入推进社区(农村)警务战略和公安、司法基层基础建设，持续强化网格化服务管理。全面落实信访工作责任制，强化社会稳定风险评估，切实有效化解各类社会矛盾纠纷。强化安全生产责任体系建设，提高重点行业、重点领域安全治理能力。推进智慧食药监管建设，严防严管严控食品药品安全风险。加强应急体系建设，夯实基层应急基础。做好国家安全、人民防空等工作。

　　提升社会文明程度。加强精神文明建设，积极倡导社会主义新风尚。坚持市县联动、全民参与，积极创建国家卫生城市、省双拥模范城，全面启动创建全国文明城市。深化群众性精神文明创建活动，创建市级文明村镇40个、文明单位30个。用好道德讲堂、市民学校等教育载体，广泛开展身边好人、最美人物等典型选评和宣传活动，切实加强公民道德教育。大力开展学雷锋志愿活动，推动志愿服务制度化、常态化，不断提升市民文明程度。

　　加强政府自身建设

　　进入新时代，肩负新使命。全市政府系统将在进一步加强法治政府、服务型政府、创新政府、廉洁政府建设基础上，着力突重点、抓关键、强机制，不断提升政府工作能力和水平，努力建设人民满意政府。

　　强化三个自觉，进一步建设政治坚定、执行坚决的政府机关。坚持把讲政治摆在政府工作的首位，坚定不移地推进政府系统政治建设。强化政治自觉。坚定政治方向，树牢“四个意识”，教育引导政府公职人员自觉维护以习近平同志为核心的党中央权威和集中统一领导，认真开展“不忘初心、牢记使命”主题教育活动，推动全市政府机关和政府工作人员自觉提高政治站位，确保政治合格、对党忠诚。强化思想自觉。推动政府工作人员解放思想，努力提升干部改革创新和组织发展的能力，坚持用市场经济思维推进全市经济社会发展，行政工作投入产出项目化，经济工作尽量数据化。强化行动自觉。坚决执行好党中央、国务院，省委、省政府的战略决策和市委工作部署，确保市委决策在政府系统落地落实，确保政府系统政令畅通。

　　突出三个重点，进一步增强依法行政能力。坚持把依法行政作为做好政府工作的基本要求。突出制度建设。建立和落实法治政府建设职责清单，协同推进城乡建设与管理、环境保护和历史文化保护领域地方立法。强化统计工作，做好第四次全国经济普查工作，确保统计数据真实可靠。突出依法决策。全面推行政府法律顾问制度，深化重大行政决策公众参与、专家论证、风险评估、合法性审查、集体讨论决定等制度。健全行政复议案件审理机制。突出权力监督。自觉接受人大及其常委会的法律监督和工作监督，落实政府重大决策出台前向人大报告制度，自觉接受政协的民主监督，认真办理人大代表建议和政协委员提案。主动听取民主党派和社会各界的意见，主动接受社会和舆论监督。强化审计监督，主动接受和支持纪检监察监督和司法监督。

正视三个问题，进一步提升政府工作效能。坚持问题导向，勇于触碰难题。着力解决“放管服”改革“肠梗阻”问题。继续优化权力事项目录，动态调整责任清单。大力推进“多证合一”“证照分离”，深化投资审批和职业资格改革，激发各类市场主体活力。加强事中事后监管，强化竞争性资源公开、公平、公正市场化配置。推广“互联网+政务服务”，形成全市一体化网上政务服务体系，实现“一号申请、一窗受理、一网通办”，推进线上线下融合发展、政务信息资源共享应用。着力解决“门好进、脸好看、事难办”的不作为、慢作为问题。建立健全马上办、受办分离、首问负责、限时办结等制度，利用现代信息手段服务社会，让数据多跑路，让群众少跑腿、少烦心。进一步改善营商环境，加大政府主动服务企业力度，年内开展纳税人对政府工作满意度测评。着力解决干部“只说不行、不想怎么行”问题。落实行政审批部门的分管领导、承办科室和主办人员责任，强化“非禁即入”审批与准入机制，鼓励干部敢于担当，促使干部既严守政策底线、又敢作敢为。今年，市政府对能办不办、观望拖办、故意刁难等庸政懒政怠政行为将坚决抓住典型，从严查处问责。

　　健全三大机制，进一步激发干部干事创业激情。坚持把激发干部动能作为当前政府工作的重要保障。建立市级部门下深水、抓项目的工作示范机制。转变市级部门工作角色，既统筹指导县区工作，又率先抓好市本级项目，以此示范带动全市工作。健全激励跳起摸高的督查考评机制。在市委统筹领导下，完善目标考核机制，健全联合督查机制，推行市政府组成部门和市属国有企业“一把手”“公开承诺、阳光考评”机制，激励干部在招商引资、项目建设等方面大胆创先争优和跳起摸高。落实容错纠错机制，为敢闯敢干、担当作为的干部撑腰鼓劲。建立网络问政和工作结果公示机制。完善政府重大事项征求意见制度，发挥“12345”政府政务服务热线作用，让民意表达更充分。建立市长与县区长、市长与市政府部门“一把手”工作微信平台，打通工作互动的“秒通道”。实行县区政府工作结果考评月通报、季登报、年终社会综合测评办法，强化责任倒逼，推进工作落实见效。坚持鼓励干部创优争先，又教育政府工作人员廉洁从政、勤奋工作。从严落实“一岗双责”，切实把全面从严治党、从严治政各项规定贯彻到政府工作各环节;认真落实中央八项规定实施细则精神和省委省政府十项规定实施细则要求，进一步深化“四风”治理;从严强化行政工作底线防控，严肃查处群众身边的不正之风和腐败问题，努力建设廉洁政府。

　　各位代表，新时代承载伟大梦想，新征程催人开拓奋进。让我们更加紧密地团结在以习近平同志为核心的党中央周围，在中共雅安市委的坚强领导下，充分依靠全市各族人民群众，团结同心、牢记使命、开拓奋进、真抓实干，努力推进全市经济社会加快发展，为建设美丽雅安、生态强市，开启我市现代化建设新征程而努力奋斗!
